# Between-hospital variation in mortality and survival after glioblastoma surgery in the Dutch Quality Registry for Neuro Surgery

**DOI:** 10.1007/s11060-019-03229-5

**Published:** 2019-06-24

**Authors:** Philip C. De Witt Hamer, Vincent K. Y. Ho, Aeilko H. Zwinderman, Linda Ackermans, Hilko Ardon, Sytske Boomstra, Wim Bouwknegt, Wimar A. van den Brink, Clemens M. Dirven, Niels A. van der Gaag, Olivier van der Veer, Albert J. S. Idema, Alfred Kloet, Jan Koopmans, Mark ter Laan, Marco J. T. Verstegen, Michiel Wagemakers, Pierre A. J. T. Robe, on behalf of the Quality Registry Neuro Surgery glioblastoma working group from the Dutch Society of Neurosurgery

**Affiliations:** 1 Department of Neurosurgery, Neurosurgical Center Amsterdam, Location VU Medical Center, Amsterdam, The Netherlands; 2 Netherlands Comprehensive Cancer Organisation (IKNL), Utrecht, The Netherlands; 3 Department of Clinical Epidemiology and Biostatistics, Academic Medical Center, Amsterdam, The Netherlands; 4 Department of Neurosurgery, Maastricht University Medical Center, Maastricht, The Netherlands; 5 Department of Neurosurgery, St Elisabeth Hospital, Tilburg, The Netherlands; 6 Department of Neurosurgery, Medical Spectrum Twente, Enschede, The Netherlands; 7 Department of Neurosurgery, Medical Center Slotervaart, Amsterdam, The Netherlands; 8 Department of Neurosurgery, Isala, Zwolle, The Netherlands; 9 Department of Neurosurgery, Erasmus University Medical Center, Rotterdam, The Netherlands; 10 HAGA Teaching Hospital, The Hague, The Netherlands; 11 Leiden University Medical Center, Leiden, The Netherlands; 12 Department of Neurosurgery, Northwest Clinics, Alkmaar, The Netherlands; 13 Department of Neurosurgery, Medical Center Haaglanden, The Hague, The Netherlands; 14 Department of Neurosurgery, Martini Hospital, Groningen, The Netherlands; 15 Department of Neurosurgery, Radboud University Medical Center, Nijmegen, The Netherlands; 16 Department of Neurosurgery, University Medical Center Groningen, University of Groningen, Groningen, The Netherlands; 17 Department of Neurology & Neurosurgery, University Medical Center Utrecht, Utrecht, The Netherlands; 18 Department of Neurosurgery, Amsterdam University Medical Centers, Location VU Medical Center, De Boelelaan 1117, 1081 HV Amsterdam, The Netherlands

**Keywords:** Glioblastoma, Neurosurgical procedures, Quality of health care, Outcome assessment, Mortality, Survival

## Abstract

**Purpose:**

Standards for surgical decisions are unavailable, hence treatment decisions can be personalized, but also introduce variation in treatment and outcome. National registrations seek to monitor healthcare quality. The goal of the study is to measure between-hospital variation in risk-standardized survival outcome after glioblastoma surgery and to explore the association between survival and hospital characteristics in conjunction with patient-related risk factors.

**Methods:**

Data of 2,409 adults with first-time glioblastoma surgery at 14 hospitals were obtained from a comprehensive, prospective population-based Quality Registry Neuro Surgery in The Netherlands between 2011 and 2014. We compared the observed survival with patient-specific risk-standardized expected early (30-day) mortality and late (2-year) survival, based on age, performance, and treatment year. We analyzed funnel plots, logistic regression and proportional hazards models.

**Results:**

Overall 30-day mortality was 5.2% and overall 2-year survival was 13.5%. Median survival varied between 4.8 and 14.9 months among hospitals, and biopsy percentages ranged between 16 and 73%. One hospital had lower than expected early mortality, and four hospitals had lower than expected late survival. Higher case volume was related with lower early mortality (P = 0.031). Patient-related risk factors (lower age; better performance; more recent years of treatment) were significantly associated with longer overall survival. Of the hospital characteristics, longer overall survival was associated with lower biopsy percentage (HR 2.09, 1.34–3.26, P = 0.001), and not with academic setting, nor with case volume.

**Conclusions:**

Hospitals vary more in late survival than early mortality after glioblastoma surgery. Widely varying biopsy percentages indicate treatment variation. Patient-related factors have a stronger association with overall survival than hospital-related factors.

**Electronic supplementary material:**

The online version of this article (10.1007/s11060-019-03229-5) contains supplementary material, which is available to authorized users.

## Introduction

The aim of glioblastoma surgery is to maximize tumor removal, while preserving the patient’s functional integrity. Because guidelines for surgical decision making are not available, treatment decisions can be highly personalized, but can introduce treatment variation and outcome variation as well. If the neurosurgeon considers a tumor unresectable, or if the patient is considered unfit or unmotivated for resective surgery, then a diagnostic biopsy can be done. More extensive tumor removal is associated with longer patient survival [1–3], whereas functional deficits from too extensive resections can result in poorer quality of life and shorter survival [4]. Before and during surgery, care teams use different techniques to optimize resections due to (1) varying access to image-guided navigation, fluorescence-guided microscopy, intraoperative MRI, or brain stimulation mapping (2) different surgical schools of education, i.c. more oncological or functional, and (3) diverging experts’ opinions on more aggressive or conservative approaches. None of these techniques has been proven to prolong patient survival.

Patient survival outcome after glioblastoma surgery varies considerably in reports from tertiary referral centers [1–3, 5–17]. Likewise, patient survival may differ among the hospitals within a nation.

The Dutch Society for Neurosurgery [18] established the Quality Registry for Neuro Surgery [19], starting with a consensus set of indicators for glioblastoma surgery in 2011. This registry provides feedback to all hospitals with neurosurgical units on clinical practice for self-assessment and quality-monitoring.

In this study, we measured variation in risk-standardized early mortality and late survival after glioblastoma surgery between all 14 hospitals that perform glioblastoma surgery in the Netherlands. Furthermore, we explored the association between survival and hospital characteristics, including case volume, academic setting and biopsy percentage, in addition to known prognostic patient characteristics, i.e. age and performance status.

## Methods

We studied all 2409 patients who had first-time surgery for glioblastoma between January 1, 2011 and December 31, 2014 at all 14 hospitals engaged in glioblastoma surgery in The Netherlands. We collected data for patients 18 years or older at surgery and a histopathological diagnosis of glioblastoma according to the WHO 2007 criteria [20].

### Data collection

Neurosurgeons, nurse specialists in neuro-oncology and trained physician assistants prospectively entered patient data in the Quality Registry for Neurological Surgery. Demographic and clinical information consisted of age at diagnosis, gender, Karnofsky performance status before surgery, type of surgery (biopsy or resection), and dates of treatment, last follow-up and death. A surgical procedure was considered a biopsy, when tissue was taken for diagnosis only, either by needle biopsy or open biopsy. For the patients who had died within one month after surgery, the cause of death was retrieved if available from the medical records or by contacting the primary care physician.

Treatment decisions for patients were made in multidisciplinary tumor board meetings in all hospitals. During resective surgery image-guided navigation was customarily used. Fluorescence-guided resection, stimulation mapping, and ultrasonography was applied by neurosurgeons’ preferences. Intraoperative MRI was not in use.

The dates of death were verified and updated against the information available from the National Cancer Registry (NCR). The NCR collects information on all newly-diagnosed cancer patients in the Netherlands following notification by the national pathology registry. Information on vital status is retrieved through yearly linkage with the Municipal Personal Records Database; on March 1, 2016 for our analyses. As a further data quality check, each hospital reviewed their data after the closure of patient inclusion.

Because this data was collected for evaluation of quality of care in accordance with the Dutch Quality Act for Healthcare [21], written informed consent was not needed. Ethical approval was waived because the study was not subject to the Medical Research Involving Human Subjects Act (WMO) [22] and de-identified data had been collected of patients mostly not alive. After delivery by a trusted third party [23], de-identified patient data was analyzed. Four authors (PWH, AZW, VHO, PRO) had full access to this data and are responsible for the data analysis and reporting.

### Outcomes and risk predictors

The main outcome measures to evaluate variation were specified in the consensus set of quality indicators of the registry: the risk-standardized early mortality and late survival. Early mortality was defined as the percentage of patients who had died of any cause within 30 days after surgery; late survival was defined as the percentage of patients who were alive at 2 years (730 days) after surgery.

To account for risk differences in glioblastoma patients among hospitals, we used known patient-related predictors for survival as covariates, i.e. age at diagnosis and Karnofsky performance status [24, 25]. We also included the year of treatment for risk-standardization, because of the four year timespan in which care decisions may have changed, although national treatment guidelines did not alter [26]. Clinical management decisions were not included in risk-standardization, such as corticosteroid use [27], surgical technique and extent of resection [1–3], and participation in clinical trials [28, 29], although associated with survival. Standard adjuvant treatment consisted of 60 Gy fractionated conformal radiotherapy with concomitant temozolomide chemotherapy, and six cycles of adjuvant temozolomide [30]. Hospital characteristics that we explored for association with outcome were the total number of patients with glioblastoma treated in 4 years in a hospital (i.e. volume), academic setting, and the percentage of biopsy procedures.

### Statistical analysis

Survival was analyzed in days with censoring at the last date of follow-up or the lookup date of alive status, and analyses were based on complete cases regarding information on covariates.

A multivariable hierarchical Cox proportional hazards model was used for risk-standardization in assessing outcome variation between hospitals. For statistical modeling and inferences, outcomes were assessed at patient-level using age, performance and year of treatment as covariates for risk-standardization. A random effect per hospital was included in the model in recognition of imperfect risk-standardization [31, 32]. Thus, for each patient, a predicted survival function was obtained based on the patient-related covariates and a hospital random effect of 0, representing treatment in a fictitious hospital with average performance. Using the patient-specific expected survival function, the standardized risk for having died within 30 days and for being alive by 730 days was calculated for each patient. In calculating risk-standardized ratios per hospital, we obtained the predicted probability of the events for each patient in a hospital and summed these probabilities to get the expected number of events for that hospital. Risk-standardized ratios per hospital were calculated as the observed number of events divided by the predicted number of events, i.e. the observed-to-expected ratio [32, 33]. For example, the risk-standardized early mortality is lower than expected with a ratio below one, and risk-standardized late survival is higher than expected with a ratio above one.

We applied a Bayesian predictive model with random effects using the Stan language [34, 35]. The counting process of events over time was assumed to follow a log-poisson density with unknown means and unknown precisions for the regression coefficients, the random effects and the hazards for which we used vague priors. Vague priors were chosen to primarily reflect inference from the presented data without substantive prior knowledge. Model details are provided as online code [36] and the predictive patient risk model as web application [37]. The posterior predictive model was verified using simulated data. No evidence against convergence was identified. The median values of posterior distributions were used as estimates with 95% credibility intervals.

Funnel plots were generated with expected number of events as precision and risk-standardized observed-to-expected event ratios per hospital as indicator as previously described [31]. The funnel control limits to identify potential outliers were obtained as 95% and 99% prediction limits from the Poisson distribution.

As a first exploration to plot regression lines between the hospital-related characteristics and observed early mortality and late survival, we used univariate logistic regression with death status at 30 days and alive status at 2 years as response measure [31]. For volume, log-transformed number of patients was modelled. Second, to estimate the effect sizes of hospital and patient-related characteristics on overall survival, we used the multivariable hierarchical Cox proportional hazards model. The hazard ratios for death were determined with 95% confidence intervals for hospital characteristics, i.e. log volume, academic setting, and biopsy percentage, and for patient-related characteristics, i.e. age, performance status, and year of treatment, as risk-standardization without hospital random effects [38].

## Results

Of the 2,409 patients, 2,308 were available for complete case analysis (Table 1). At last follow-up, 462 patients were alive.

**Table 1:** Characteristics of patients and hospitals with survival outcome per hospital and overall

Hospital	a	b	c	d	e	f	g	h	i	j	k	l	m	n	Overall
Number of patients	81	229	293	97	161	269	197	91	103	233	103	121	73	358	2409
No. complete case analysis	77	228	277	95	116	268	196	82	102	232	103	111	73	348	2308
Patient characteristics															
Gender															
No. male	49	152	178	61	97	172	127	58	55	145	64	55	44	219	1476
No. female	32	77	114	36	63	94	70	33	48	88	39	47	29	136	906
Age															
Age, mean, years	64.6	60.5	63.6	54.6	60.3	60.6	60.8	62.9	61.2	61.7	64.8	59.7	59.0	62.0	61.4
Age, SD, years	10.8	13.3	11.9	14.7	11.9	12.6	12.2	10.2	12.5	11.8	10.8	12.7	13.0	10.9	12.2
No. missing age	1	0	0	1	0	0	1	0	0	1	0	0	0	6	10
Karnofsky performance scale															
No. KPS 100	1	23	9	35	3	25	15	8	1	12	10	3	16	55	216
No. KPS 90	30	57	91	25	44	82	56	26	50	84	26	28	24	167	790
No. KPS 80	21	48	65	19	30	62	47	26	27	87	38	33	11	67	581
No. KPS 70	8	59	54	13	21	54	25	10	9	26	16	21	10	31	357
No. KPS 60	13	29	33	1	11	23	24	3	10	15	8	4	6	19	199
No. KPS 50	4	11	19	1	3	12	20	11	6	5	5	11	4	15	127
No. KPS 40	0	1	4	2	0	2	3	2	0	2	0	4	1	0	21
No. KPS 30	0	0	1	0	1	2	6	1	0	0	0	1	1	0	13
No. KPS 20	1	0	1	0	3	5	0	3	0	2	0	7	0	0	22
No. KPS 10	0	0	0	0	0	1	1	0	0	0	0	0	0	0	2
No. KPS missing	3	1	16	1	45	1	0	1	0	0	0	9	0	4	81
Year of treatment															
No. 2011	9	53	3	23	42	66	58	31	20	63	21	26	13	95	523
No. 2012	23	49	99	16	46	68	45	20	30	63	35	2	14	91	601
No. 2013	21	53	102	25	26	58	47	24	27	56	29	37	26	78	609
No. 2014	28	74	89	33	47	77	47	16	26	51	18	56	20	94	676
Hospital characteristics															
Academic setting	Yes	Yes	Yes	Yes	Yes	Yes	Yes	No	No	No	No	No	No	No	7
Surgery															
No. resection	60	175	192	23	49	187	154	65	72	179	44	102	53	209	1564
No. biopsy	21	54	101	62	42	68	43	25	31	54	58	19	20	147	745
Biopsy percentage	25.9%	23.6%	34.5%	72.9%	46.2%	26.7%	21.8%	27.8%	30.1%	23.2%	56.9%	15.7%	27.4%	41.3%	32.3%
Survival outcome															
Overall survival, median, months	10.2	11.4	10.0	14.9	7.4	10.8	9.5	10.3	5.2	10.7	4.8	12.0	12.1	10.3	10.2
Early mortality															
No. observed deaths at 30 days	6	6	18	4	10	10	10	6	6	9	6	11	3	14	119
No. of observable patients at 30 days	77	228	277	95	116	268	196	82	102	232	103	111	73	348	2308
Mortality at 30 days	7.8%	2.6%	6.5%	4.2%	8.6%	3.7%	5.1%	7.3%	5.9%	3.9%	5.8%	9.9%	4.1%	4.0%	5.2%
Late survival															
No. observed survivors at 2 years	3	36	39	18	11	30	28	7	0	32	5	16	8	37	270
No. of observable patients at 2 years	67	210	255	78	105	236	183	78	12	211	91	93	65	317	2001
Survival at 2 years	4.5%	17.1%	15.3%	23.1%	10.5%	12.7%	15.3%	9.0%	0.0%	15.2%	5.5%	17.2%	12.3%	11.7%	13.5%

**Fig. 1:**
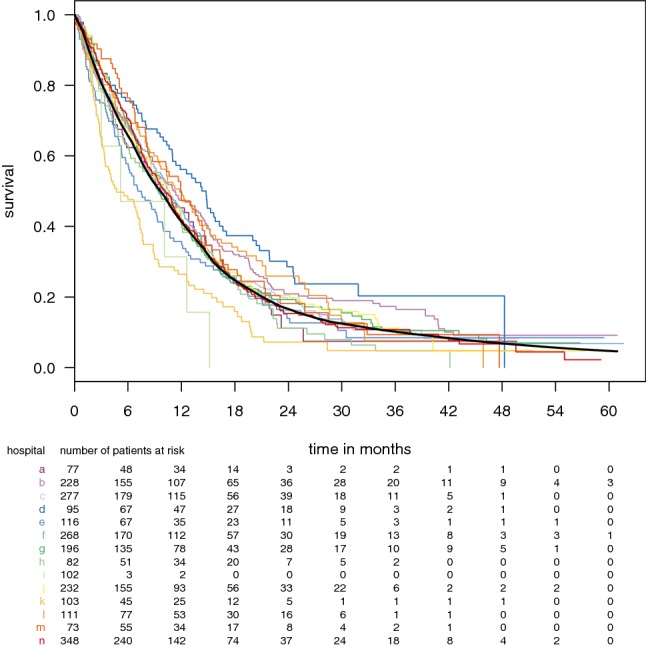
Survival outcome over months as Kaplan–Meier curves per hospital in colors and overall survival function in black based on the Cox regression model with risk-standardization for age, performance, and treatment year. The hospital identifications correspond with Table 1

The observed overall survival over time per hospital and the risk-standardized survival function for all patients are plotted in Fig. 1. The observed and expected survival over time per hospital is shown in Supplemental Fig. 1.

The overall 30-day mortality was 5.2% and the overall 2-year survival was 13.5%. The observed early mortality and late survival for each hospital is listed in Table 1. Median overall survival was 10.2 months, and varied between 4.8 and 14.9 months among hospitals.

**Fig. 2:**
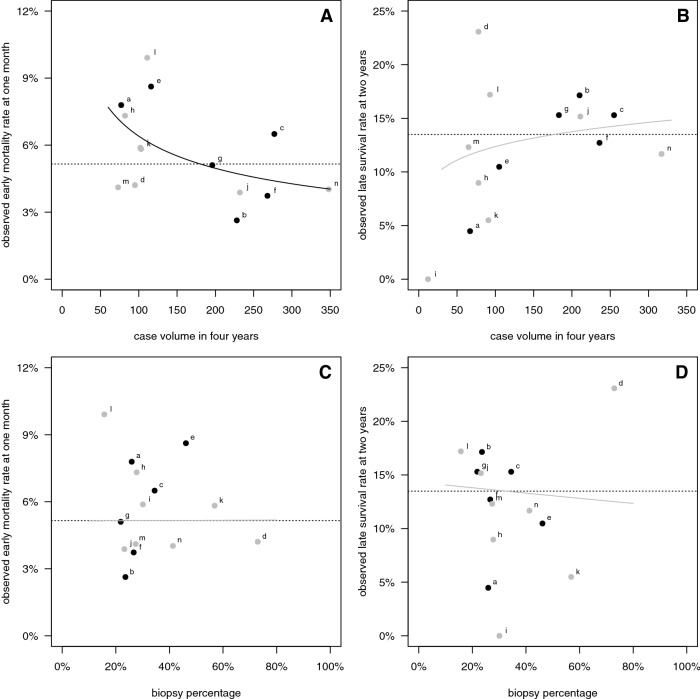
Hospital characteristics versus survival outcome. Plots of **a** case volume in 4 years versus observed early mortality percentage, **b** volume versus observed late survival percentage, **c** biopsy percentage versus observed early mortality percentage, and **d** biopsy percentage versus observed late survival percentage. Black circles indicate hospitals with an academic setting. The overall outcome percentages are represented by dotted lines. Logistic regression lines are drawn, significant association estimates in black, non-significant estimates in grey

The hospital characteristics of case volume, academic setting and biopsy percentage are plotted in relation to observed early mortality and late survival in Fig. 2. Case volume varied between 73 and 358 patients in 4 years. In univariate logistic regression analysis, a higher volume was related with lower early mortality (P = 0.031), but not with late survival. The estimated log OR of log volume on early mortality was − 0.39, so that a 10% increase in volume was estimated to be associated with 3.9% relative decrease in early mortality. The estimated boundary from higher-than-average to lower-than-average early mortality is located at a volume of about 180 patients in 4 years, i.e. 45 patients per year. In this first univariable exploration, an academic setting of hospitals was not significantly associated with early mortality or late survival. The biopsy percentage varied between 16 and 73% among hospitals, indicating considerable treatment variation. The biopsy percentage was not significantly associated with early mortality or late survival in this first exploration.

In our effort to retrieve the causes of early death, information was obtained for all 119 patients who had died within 30 days. Early death was related to glioblastoma progression in 36 (30%) patients, the cause remained unknown in 36 (30%), death was directly related to surgery in 31 (26%), i.e. hemorrhage in 16, postresection edema and/or ischemia in seven, postoperative functional deterioration in seven, and intracranial infectious complications in three. Early death could be indirectly related to surgery in 13 (11%), i.e. seizures in five, pulmonary embolus in five, and extracranial infectious complications in three, and early death was unrelated to either the disease or surgery in three (3%), i.e. cardiac arrest in two, and trauma in one.

**Fig. 3:**
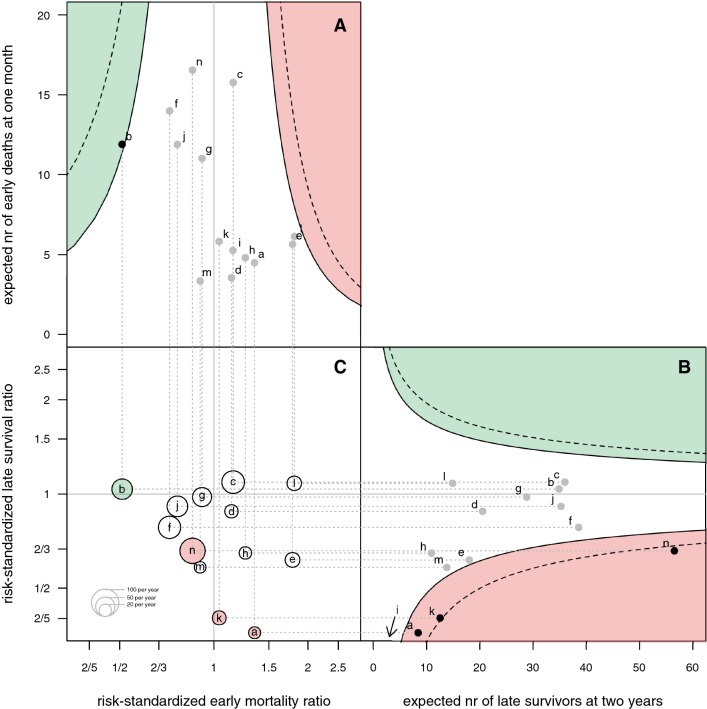
Multipanel plot of expected numbers of early deaths versus risk-standardized mortality ratios (**a**), expected numbers of late survivors versus risk-standardized late survival ratios (**b**), and combination plot of risk-standardized early mortality versus late survival ratios on log scales (**c**). The solid funnels are 95% control limits, the dotted funnels 99% control limits. Black dots indicate hospitals with ratios outside the 95% control limits. Better than expected early mortality and late survival is shown in green, worse than expected is shown in red. The institutional identifications are printed in the circles; sizes correspond with the case volumes according to the legend. Note that hospital i has 0 observed late survivors and 0.89 expected late survivors, which therefore is outside the plot below the lower 99% control limit and in the red region

From the funnel plots in Fig. 3, one hospital (b) had lower early mortality than expected within 95% control, and four hospitals (a, i, k, and n) had lower late survival than expected within 95% control. The combination plot of early mortality and late survival indicates that the two outcomes are not related. In other words, this indicates that more extensive surgery for longer tumor control is not set off by an increase in postoperative mortality.

In the multivariable Cox regression analysis, the known prognostic factors of older age (HR 1.54, 1.46–1.62, P < 0.00001) and worse performance (HR 0.77, 0.74–0.81, P < 0.00001) were strongly associated with shorter survival, as expected. More recent years of treatment were also associated with longer survival with 2011 as reference (2012 HR 0.94, 0.83–1.07, NS; 2013 HR 0.80, 0.71–0.92, P = 0.001; 2014 HR 0.78, 0.68–0.89, P = 0.0003). Therefore, risk-standardization with these patient-related characteristics seems justified. Of the hospital characteristics, a lower biopsy percentage was associated with longer overall survival (HR 2.09, 1.34–3.26, P = 0.001). Log case volume (HR 0.954, 0.866–1.05) and academic setting (HR 0.951, 0.858–1.05) were not associated with overall survival.

## Discussion

This comprehensive, nation-wide, four-year prospective quality registry study on survival outcomes after glioblastoma surgery shows (1) between-hospital variation in 2-year survival and 30-day mortality (2) surgical treatment variation suggested by widely-varying biopsy percentages between hospitals (3) that 30-day mortality is not a suitable measure for glioblastoma surgery-related complications only, as many of these patients die early from progressive disease (4) that a larger case volume is associated with lower early mortality, but not with overall survival (5) that in addition to patient-related factors, i.e. younger age and better performance, a lower biopsy percentage in a hospital is an important indicator of overall survival outcome, and not case volume nor an academic setting.

Differences in late survival after glioblastoma surgery between hospitals have not been published. The 2-year survival of glioblastoma in our overall data is comparable to other registries, i.e. 14.8% in the Central Brain Tumor Registry of the United States [39] and 23.8% in the Surveillance, Epidemiology and End Results Program of the National Cancer Institute [40], and comparable to other communities, ranging from 12.0 to 25.3% [9, 15–17]. Registry-based populations include patients typically excluded from clinical trials and surgical resection series, such as elderly patients with treatment concessions, resulting in shorter survival in real-life data than reported in clinical trials.

Early mortality differences between hospitals have been reported earlier, indicating that high-volume surgeons in high-volume hospitals have lower in-hospital mortality and complication rates after brain tumor surgery based on the Nationwide Inpatient Sample data of 62,514 admissions [41]. Similarly, high-volume hospitals had lower mortality after brain tumor resections based on a state-based study of 4,723 patients in 33 hospitals [42]. Postoperative mortality estimates for brain tumor surgery have varied considerably: 0.26% in 8,091 patients based on a meta-analysis of 90 publications [43], 1.0% in 306 patients from one hospital [4], 1.5% in 408 patients from 52 hospitals [44], 1.7% in 400 patients from one hospital [45], 2.5% in 322 patients in a multicenter randomized trial [46], 3.5% in 4,723 patients from 33 hospitals [42], and 7.9% in 834 patients from 19 hospitals [9]. Variability in these estimates may be explained by different timings of mortality, inclusion of tumors other than glioblastoma, patient selection bias, and publication bias. Our results indicate that mortality within 30 days is not a useful quality indicator for glioblastoma surgery-related complications without information on causes of death. A more precise measure would be the percentage of surgery-related mortality.

Earlier reports have identified an association between larger case volumes and more favorable outcome after glioblastoma surgery [41, 42, 47] and after other cancer-related surgery [48, 49]. In our findings, larger case volume is not associated with overall survival when adjusted for patient-related risk factors. Patient-related factors clearly outweigh hospital-related effects. A prominent hospital-related predictor of overall survival in our data is the percentage of biopsies. One explanation is that higher extent of resection has been shown to prolong patient survival [1–3]. In addition, we speculate that the percentage of biopsies may be a surrogate marker for a more conservative general approach with possibly earlier cessation of therapies to rescue or to prolong life. The biopsy percentages among hospitals varied considerably, whereas the patient risk profiles of hospitals were quite similar. The causes for the biopsy percentage variation and MRI-based glioblastoma removal measurements need to be explored as quality indicators in further studies. This should enhance exchange of team expertise and surgical skills. Other quality indicators for glioblastoma surgery may include functional outcome, measured as patient-related outcome measures, cognitive performance, or health-related quality of life. Furthermore, the National Surgical Quality Improvement Program recently reported on hospital process measures, such as length of hospital stay [50], readmission rate [51], and unplanned reoperation [52].

Our results highlight the importance of risk-standardization in comparing hospital outcomes [31, 32]. For example, hospital ‘d’ has the highest observed percentage of 2-year survival without risk-adjustment (23% in Table 1), whereas the 2-year survival ratio adjusted for patient-related risk factors is less than expected within control limits (0.88 in Fig. 3b). This can be explained by a patient population that is on average younger and has better performance than populations of other hospitals (Table 1). This may indicate a deviating patient referral or selection in hospital ‘d’.

Survival time is an objective measure. The interpretation, however, of early mortality or late survival as success or failure of treatment decisions is not necessarily straightforward. For example, a patient not alive at 30 days may have died from rapidly progressive disease despite optimal treatment decisions, whereas a patient alive at 2 years may be in a poor condition with minimal quality of life for a prolonged period of time from overtreatment. Therefore, a spectrum of quality indicators is required to capture the nuances of quality of care.

The yearly feedback of our registry and the results of this analysis have resulted in discussions on quality-monitoring within our workgroup and in self-assessment by neurosurgeons within departments. This collaboration has been perceived as constructive and encouraging. The observation that more recent treatment years were associated with longer overall survival may indicate a quality improvement as a result of this collaboration.

The strengths of our study are the comprehensive population-based nation-wide cohort, the data quality checks from two data sources (QRNS and NCR), the near-complete follow-up of patients, and the modern methods for prediction modeling. The limitations of our study are the unavoidable imperfect patient-level risk-standardization, the unavailability of information - other than biopsy or resection - on applied surgical techniques and the unavailability of other hospital characteristics, such as treatment guideline adherence or clinical trial participation. Furthermore, the few patients who refrained from any treatment or who had radiation or chemotherapy without histopathological diagnosis were not included in this pathology-based registry. Similarly, the few patients, who may have crossed over between hospitals for treatment, remain unidentified. And treatment variation other than surgical decisions may contribute to the observed outcome variation. For this, we have recently started a national multidisciplinary collaboration involving radiation and medical oncologists, neurologists, radiologists, and pathologists for a joined quality registry, i.e. the Dutch Brain Tumor Registry.

There are several possible implications for clinical practice. First, a quality program is required to enforce hospitals with less than expected outcome to improve, for instance by identification of differences in care programs amenable to exchange expertise, and to ultimately devise a systematic quality evaluation and improvement cycle. At the same time regionalization of brain tumor care in networks may improve overall quality of care. Second, further investigation is necessary into the relation between hospital biopsy percentage, volume, and survival outcome. It remains undetermined whether a threshold for minimum case load per year is a robust criterium for ‘in control’ survival outcome. Third, early mortality should be reported with causes of death in quality comparisons. Fourth, patient counseling and surgical decision making should rather be based on personalized predictions from real practice data than on clinical trial results and tertiary referral center publications [53]. Therefore, our predictive patient risk model may be useful in clinical decision making [37].

## Conclusion

Hospitals vary more in late survival than early mortality after glioblastoma surgery. Widely varying biopsy percentages indicate treatment variation. Patient-related factors have a stronger association with overall survival than hospital-related factors.

## Electronic supplementary material

Below is the link to the electronic supplementary material.

Supplemental Figure 1 Survival outcome per hospital over months after surgery. The observed survival is plotted in thick black as Kaplan-Meier curve with censoring of patients; the expected hospital survival based on risk-standardization for patient characteristics is plotted as survival function in blue. The overall survival function is shown as reference in grey dots. The fitted survival using the hospital-specific random effect for prediction is shown as thin black curve. Vertical lines are drawn at one month and at two years. The last two plots show the deviation between observed and expected number of deaths per hospital, as absolute difference and as ratio, respectively (DOCX 2606 kb)
