# Supplementary material for: Between-hospital variation in mortality and survival after glioblastoma surgery in the Dutch Quality Registry for Neuro Surgery
Source: J Neurooncol. 2019 Jun 24;144(2):313–23. doi: 10.1007/s11060-019-03229-5 (PMC6700042; doi:10.1007/s11060-019-03229-5)

Supplementary figure 1.

Survival outcome per hospital over months after surgery. The observed survival is plotted in thick black as Kaplan-Meier curve with censoring of patients; the expected hospital survival based on risk-standardization for patient characteristics is plotted as survival function in blue. The overall survival function is shown as reference in grey dots. The fitted survival using the hospital-specific random effect for prediction is shown as thin black curve. Vertical lines are drawn at one month and at two years. The last two plots show the deviation between observed and expected number of deaths per hospital, as absolute difference and as ratio, respectively.


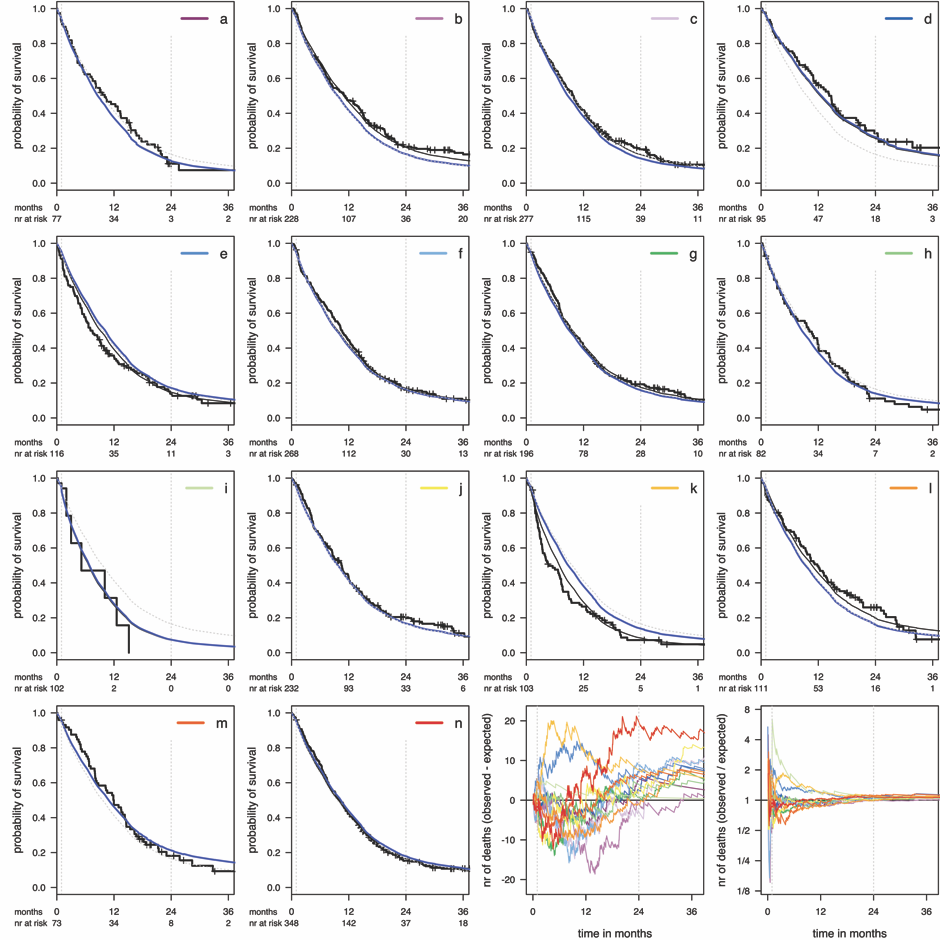

Supplement: Supplementary file 1 — Supplemental Figure 1 Survival outcome per hospital over months after surgery. The observed survival is plotted in thick black as Kaplan-Meier curve with censoring of patients; the expected hospital survival based on risk-standardization for patient characteristics is plotted as survival function in blue. The overall survival function is shown as reference in grey dots. The fitted survival using the hospital-specific random effect for prediction is shown as thin black curve. Vertical lines are drawn at one month and at two years. The last two plots show the deviation between observed and expected number of deaths per hospital, as absolute difference and as ratio, respectively (DOCX 2606 kb) [file 11060_2019_3229_MOESM1_ESM.docx]
